# Dual-targeting of aberrant glucose metabolism in glioblastoma

**DOI:** 10.1186/s13046-015-0130-0

**Published:** 2015-02-05

**Authors:** Han Shen, Stephanie Decollogne, Pierre J Dilda, Eric Hau, Sylvia A Chung, Peter P Luk, Philip J Hogg, Kerrie L McDonald

**Affiliations:** Cure Brain Cancer Neuro-Oncology Group, Adult Cancer Program, Lowy Cancer Research Centre and Prince of Wales Clinical School, University of New South Wales, Sydney, 2052 Australia; Tumour Metabolism Group, Adult Cancer Program, Lowy Cancer Research Centre and Prince of Wales Clinical School, University of New South Wales, Sydney, NSW 2052 Australia; Cancer Care Centre, St George Hospital, Kogarah, NSW 2217 Australia

**Keywords:** Cancer, Metabolism, PENAO, Dichloroacetate, Apoptosis

## Abstract

**Background:**

Glioblastoma (GBM) is the most common and malignant primary brain tumor. In contrast to some other tumor types, aberrant glucose metabolism is an important component of GBM growth and chemoresistance. Recent studies of human orthotopic GBM in mice and *in situ* demonstrated GBM cells rely on both glycolysis and mitochondrial oxidation for glucose catabolism. These observations suggest that the homeostasis of energy metabolism of GBM cells might be further disturbed by dual-inhibition of glucose metabolism. The present study aimed to evaluate the efficacy and the mechanisms of dual-targeting therapy in GBM cells.

**Methods:**

Representative GBM cells (immortalized GBM cell lines and patient-derived GBM cells) and non-cancerous cells were treated with 4-(N-(S-penicillaminylacetyl)amino) phenylarsonous acid (PENAO), an in-house designed novel arsenic-based mitochondrial toxin, in combination with dichloroacetate (DCA), a pyruvate dehydrogenase kinase inhibitor. The efficacy of this combinatorial therapy was evaluated by MTS assay, clonogenic surviving assay and apoptotic assays. The underlying mechanisms of this dual-targeting treatment were unraveled by using mitochondrial membrane potential measurements, cytosol/mitochondrial ROS detection, western blotting, extracellular flux assay and mass spectrometry.

**Results:**

As monotherapies, both PENAO and DCA induced proliferation arrest in a panel of GBM cell lines and primary isolates. PENAO inhibited oxygen consumption, induced oxidative stress and depolarized mitochondrial membrane potential, which in turn activated mitochondria-mediated apoptosis. By combining DCA with PENAO, the two drugs worked synergistically to inhibit cell proliferation (but had no significant effect on non-cancerous cells), impair the clonogenicity, and induce mitochondria-mediated apoptosis. An oxidative stress of mitochondrial origin takes a prominent place in the mechanism by which the combination of PENAO and DCA induces cell death. Additionally, PENAO-induced oxidative damage was enhanced by DCA through glycolytic inhibition which in turn diminished acid production induced by PENAO. Moreover, DCA treatment also led to an alteration in the multidrug resistance (MDR) phenotype of GBM cells, thereby leading to an increased cytosolic accumulation of PENAO.

**Conclusions:**

The findings of this study shed a new light with respect to the dual-targeting of glucose metabolism in GBM cells and the innovative combination of PENAO and DCA shows promise in expanding GBM therapies.

## Background

Glioblastoma (GBM) is the most common and malignant primary brain tumor in adults [1]. Despite significant improvements in the multimodality treatment, tumor recurrence is inevitable and the overall survival is dismal with a median survival of only 15 months [1]. New therapeutic strategies and combinations are urgently needed.

Proliferating cancer cells preferentially utilize glycolysis to support growth. This metabolic alteration is commonly referred to as the Warburg effect. GBM, like most cancers, presents this unique metabolic state to utilize glycolysis for ATP generation [2]. Due to the disparity between the metabolism of cancer and normal cells, several agents that specifically inhibit glycolysis have been used as anticancer agents both *in vitro* and *in vivo* [3-5]. Another prominent feature of cancer cells is their persistent resistance to mitochondria-mediated apoptosis leading to immortalization [2,6]. Mitochondria play a central role in ATP production and are also involved in a wide array of cellular processes such as cell metabolism, proliferation and cell death [7]. Therefore, targeting tumor mitochondria is a highly attractive antitumor therapeutic strategy [8].

That both glycolysis and mitochondrial oxidative phosphorylation (OXPHOS) play key roles in cancer cells has led to a new research focus into drugs that inhibit both pathways [7,9,10]. Dichloroacetate (DCA), a pyruvate dehydrogenase kinase (PDK) inhibitor that reverses the Warburg effect [11,12], has been demonstrated to inhibit tumor growth *in vivo* [11,13,14], and induce apoptosis in tumors of GBM patients by normalizing the mitochondrial activity [15]. In cancer treatment, the mechanism by which DCA induces apoptosis of cancer cells is via an enhancement of a flux of electrons through the electron transport chain (ETC.) resulting in greater depolarization of the mitochondrial membrane potential (which is generally hyperpolarized in tumor cells) and release of cytochrome c followed by subsequent activation of apoptosis [11]. However, there are some conflicting reports for DCA’s anti-tumor efficacy *in vitro* and *in vivo* [16]. In particular, not all studies found induction of apoptosis with DCA alone at clinical relevant concentrations when tested *in vitro* [17]. Improved sensitization of tumor cells to glycolysis inhibition has been achieved by the combination of glycolytic inhibitors and mitochondrial toxins [18,19]. As a glycolytic inhibitor, DCA has also been reported to be more effective when combined with mitochondria-targeted agents [20,21]. Specifically, DCA has been demonstrated to sensitize cancer cells towards apoptosis and enhance the effects of several anti-cancer agents, including arsenic trioxide [20], cisplatin [22,23] and metformin [24]. In this way, dual targeting of glucose metabolism, using DCA to restore suppressed mitochondrial activity and then an anti-mitochondrial agent to simultaneously inhibit mitochondrial function, is a rational strategy to eradicate immortalized cancer cells by disturbing their bioenergetic metabolism.

4-(N-(S-penicillaminylacetyl)amino) phenylarsonous acid (PENAO) is an in-house designed second generation arsenic-based mitochondrial toxin that is being tested in a Phase I dose escalation trial in patients with solid tumors refractory to standard therapy. PENAO inactivates adenine nucleotide translocase (ANT), a component of the mitochondrial permeability transition pore (MPTP) located in the inner-mitochondrial membrane, thereby triggering mitochondrial apoptotic pathway [25,26]. The trivalent arsenical moiety of PENAO reacts with ANT, crosslinking Cys^57^ and Cys^257^ of ANT to trigger MPTP opening by increasing the sensitivity of pore opening to Ca^2+^ levels [26]. PENAO is taken up into cells faster and its export by multidrug resistant (MDR) protein 1 and 2 (MRP1/2) is slower compared to its previous generation, 4-(N-(S-glutathionylacetyl)amino)phenylarsenoxide (GSAO) [27], enabling it to target both proliferating tumor and endothelial cells. PENAO has been demonstrated to inhibit proliferation of a range of cancer cell lines as well as endothelial cells *in vitro* [25], and is also effective *in vivo* against subcutaneous human BxPC-3 pancreatic carcinoma xenografts [25].

The present study examines the anti-tumor activity and interplay of PENAO and DCA, two metabolism targeting drugs. We demonstrated that DCA enhanced the cytotoxicity of PENAO to GBM cells through a mechanism involving GSH-mediated redox changes while simultaneously offsetting the acid production induced by PENAO, potentially providing a dual therapeutic advantage.

## Methods

### Cell culture and chemicals

GBM cell lines (U87, U251, LN229, DBTRG) and non-cancerous human lung fibroblast cell line (MRC-5) were purchased from ATCC. U87, U251 and MRC-5 were cultured in MEM (Gibco) with 10% fetal bovine serum (FBS) and 2 mM L-glutamine. LN229 was cultured in DMEM (Gibco) with 10% FBS and 2 mM L-glutamine. DBTRG was cultured in RPMI1640 (Gibco) supplemented with 10% FBS, 2 mM L-glutamine, 25 mM HEPES and 1 mM sodium pyruvate. Patient-derived GBM cell line BAH1 was kindly provided by our collaborative researchers at Queensland Institute of Medical Research and was cultured in Advanced DMEM/F12 (Gibco) mixed with Neurobasal^TM^-A medium (Gibco) (1:1) supplemented with B-27 (1×), FGF (20 ng/mL) and EGF (20 ng/mL). Normal human astrocytes were purchased from Lonza and cultured in Astrocyte Growth Medium with Astrocyte Medium Bullet Kit (Lonza). PENAO was synthesized as previously published [25]. Sodium dichloroacetate (DCA), N-acetyl-L-cysteine (NAC), glutathione reduced ethyl ester (GSH-MEE) and buthionine sulphoximine (BSO) were purchased from Sigma-Aldrich.

### Cell proliferation assay

Cells were seeded in 96-well plates followed by treatments with PENAO, DCA, or PENAO-DCA combination for 72 hr. Cell viability was determined using MTS assay (Promega) according to the manufacturer’s instructions.

### Cell cycle analysis

Cells were seeded in 6-well plates, followed by treatments as indicated. After treatments, cells were harvested and fixed in cold 70% v/v ethanol for at least 2 hr. Fixed cells were washed with phosphate-buffered saline (PBS) and stained in the dark with a solution containing propidium iodide (PI) (10 μg/mL), Triton X-100 (0.1%) and RNAse (100 μg/mL) for 20 min at room temperature. DNA content was analyzed using a BD FACSCanto II flow cytometer and data analysis was performed using FlowJo (TreeStar Inc).

### Colony formation assay

Cells were seeded into 6-well plates followed by treatments as indicated. Culture medium was changed after 24 hr treatment. Plates were incubated for 2 weeks undisturbed. Colonies were gently washed 2× with PBS followed by staining and fixation with crystal violet solution (0.5% in H_2_O: Methanol 1:1) for 15 min; the crystal violet solution was removed, and the plates were washed by immersing in a bucket of cold tap water until the water ran clear; plates were then inverted on an absorbent pad and allowed to dry overnight. Stained colonies consisting of >50 cells were counted and the number was recorded.

### Apoptotic assay

Apoptosis was quantified by Annexin-V-FLUOS Staining Kit (Roche) according to the manufacturer's instructions. Briefly, cells were seeded in 6-wells plates, followed by treatments as indicated for 24 hr. Cells were harvested and stained with a solution containing both Annexin-V and PI for 30 min, followed by flow cytometry analysis. As a second confirmation of apoptosis, cleaved Poly (ADP-ribose) polymerase (c-PARP) was detected by western blotting.

### Mitochondrial membrane potential assay

Cells were plated in 6-well plates as above followed by treatments as indicated for 16 hr. JC-1 (Sigma; 2 μM) was added and the cells were allowed to incubate in the stain for 15 min in the dark at 37°C. Cells were then trypsinized, centrifuged, washed with PBS and resuspended in the same buffer (500 μL). JC-1 fluorescence was measured using flow cytometer.

### Cytosol/mitochondrial ROS detection

Cells were seeded in 6-well plates, followed by treatments as indicated for 6 hr. Dihydroethidium (DHE) (Invitrogen) and MitoSOX Red (Invitrogen) were used to measure the level of cytosol and mitochondrial ROS production respectively according to the manufacturer’s instructions. After staining, cells were trypsinized, centrifuged and resuspended in 1 mL PBS (for DHE) or Hank’s Balanced Salt Solution (HBSS) (for MitoSOX). Sytox Blue (Invitrogen; 1 μM) was added to counter-stain for non-viable cells. DHE and MitoSOX Red fluorescence was analyzed using flow cytometer.

### Western blotting

Cells were seeded in 6-well plates, followed by treatments as indicated. Cells were lysed with RIPA buffer (150 mM NaCl, 1% Triton X-100, 0.5% sodium deoxycholate, 0.1% SDS, 50 mM Tris, pH 8.0), sonicated and centrifuged (14000 rpm, 10 min, 4°C). Supernatant protein content was measured using BCA Assay Kit (Pierce, Rockford, IL, USA). Proteins (50 μg/sample) were separated via reducing 10% SDS-PAGE and standard western blotting procedures [28] were used to detect proteins of interest with the following primary antibodies: c-PARP (Cell Signaling Technology, #9541), γ-H2AX (Cell Signaling Technology, #9718), MRP1 (Enzo Life Sciences, #106-80107), β-actin (Abcam, ab8227).

### Detection of cytosolic PENAO accumulation

Cells were seeded in 6-well plates at a density of 7.5 × 10^5^ cells (U87) or 1 × 10^6^ cells (BAH1) per well and allowed to attach for 24 hr, The cells were treated with 50 μM PENAO for 4 hr at 37°C, 5% CO2. When indicated, before the addition of PENAO the cells were pre-treated with 20 mM DCA for 24 hr. After 4 hr of contact with PENAO, the culture medium was removed and the cells were washed twice with ice-cold PBS (5 ml each time). Cells were left for drying overnight and then lysed with 1 ml of 70% w/w nitric acid. Lysates were diluted 50-fold and analyzed for arsenic atoms using an Elan 6100 Inductively Coupled Plasma Spectrometer (PerkinElmer Sciex Instruments). An identical plate was set up in the same experimental setting for protein extraction. Protein concentration was measured using the Pierce BCA Protein Assay, and the arsenical level was normalized to protein content of the samples.

### Extracellular flux assay

Cells were seeded in Matrigel-coated XF24 Seahorse Bioscience cell culture plates at 3 × 10^4^ cells (U87) or 5× 10^4^ cells (BAH1) cells per well in culture media for 24 hr, followed by treatment with indicated drug concentrations for another 24 hr. After 48 hr, cell medium was changed to unbuffered DMEM containing the same treatment as the previous 24 hr. After calibration of the XF24 sensor cartridge, the cell plate was loaded in the analyzer followed by measurements with oxygen consumption rate (OCR) and extracellular acidification rate (ECAR) over 1 hr. At the end of the assay, cells were harvested and the numbers of viable cells were determined by flow cytometry. The measurements of OCR and ECAR were normalized using the viable cell count.

### Statistical analyses

All analyses were performed using GraphPad Prism. Each independent experiment was performed with at least triplicate samples per treatment group. Results are expressed either as mean ± s.d. of replicate values from three independent experiments or representative of three independent experiments expressed as means ± s.d. of replicate measurements. Statistical analysis was performed by two-way analysis of variance corrected by Dunnet’s test or Student’s t test. All tests of statistical significance were two-sided and p values < 0.05 were considered statistically significant.

## Results

### PENAO in combination with DCA inhibits the growth of GBM cells *in vitro*

**Figure 1:**
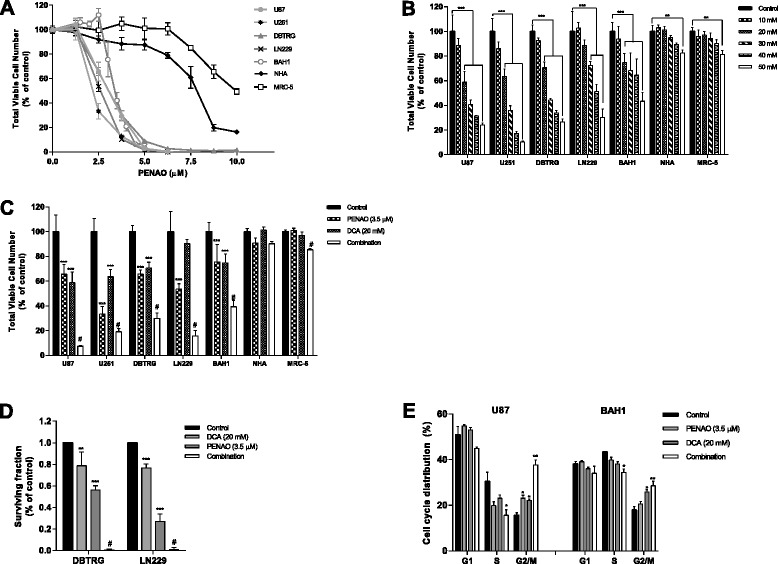
**Combining DCA with PENAO inhibited the growth of GBM cells** ***in vitro*** **. (A)** Anti-proliferation effect of PENAO on a panel of GBM cells and non-cancerous cells after 72 hr treatment. **(B)** Viable cell number after 72 hr DCA treatment. **(C)** Viable cell number after 72 hr treatment of 3.5 μM PENAO, 20 mM DCA and their combination. **(D)** The clonogenicity of GBM cells (DBTRG and LN229) treated with PENAO, DCA and the combination. **(E)** Cell cycle distribution of GBM cells (U87 and BAH1) after 24 hr treatment with PENAO, DCA and PENAO-DCA combination. Results are presented as means ± s.d. of three experiments. *p < 0.05, **p < 0.01, ***p < 0.001, #p < 0.01 vs PENAO.

To examine the effects of PENAO-DCA combination on GBM cells, we measured cell proliferation and anchorage-dependent colony formation. Both GBM and the non-cancerous cell lines were treated with PENAO and DCA for 72 hr at different concentrations. Inhibition of proliferation was measured and the half-maximal inhibitory concentrations (IC_50_) of both drugs were determined. The IC_50_ values of PENAO-induced proliferation arrest were at low micromolar range of 2.5-3.5 μM for all tested GBM cell lines (Figure 1A and Table 1). In contrast, the IC_50_ values of DCA were at suprapharmacological millimolar level (Figure 1B and Table 1). The IC_50_ values of PENAO for non-cancerous cells were 2–3 times higher than that for GBM cells, whilst the IC_50_ values of DCA for non-cancerous cells were not reached (DCA concentration in this study was tested up to 50 mM) (Figure 1A,B and Table 1). The combinations of 3.5 μM PENAO and 20 mM DCA were tested under the same experimental conditions and cell viability was measured (Figure 1C). The PENAO-DCA combination further reduced cell viability in all GBM cells compared to each drug alone but had no significant effect on non-cancerous cells (Figure 1C). To confirm the effects observed on cell proliferation, we employed an anchorage-dependent liquid colony culture for 2 weeks, at the end of which the number of colonies was quantified. Consistently, either 3.5 μM PENAO or 20 mM DCA reduced the number of colonies in both DBTRG and LN229 cells (Figure 1D). A significant inhibition of clonogenicity occurred when cells were treated with PENAO-DCA combination (Figure 1D).

**Table 1:** **Cell viabilities of GBM cells and non-cancerous cells after treatments with PENAO, DCA and combination**

**Compounds**	**IC**_**50**_ **values**						
	**GBM cell lines**					**Non-canerous cell lines**	
	**U87**	**U251**	**DBTRG**	**LN229**	**BAH1**	**MRC-5**	**Normal human astrocytes**
**PENAO (** **μM)**	3.5 ± 0.5	2.5 ± 0.4	3.5 ± 0.2	3.0 ± 0.4	3.4 ± 0.3	8.7 ± 0.7	7.9 ± 1.2
**DCA (mM)**	25.6 ± 6.2	24.3 ± 5.7	28.2 ± 4.5	40.3 ± 5.2	46.5 ± 6.2	n.d.	n.d.

The half maximal inhibitory concentration (IC_50_) values were determined by MTS assay at 72 hr time point for all drug treatments. Values are mean ± s.d. of three independent experiments, performed in triplicates. Cells were grown as adherent monolayers. n.d. = not determined.

### The combination of PENAO and DCA induces G2/M phase cell cycle arrest

We investigated the PENAO-DCA effect on cell cycle as a possible cause of the decreased growth rate observed above. U87 cells were treated with 3.5 μM PENAO and 20 mM DCA for 24 hr, and cell cycle profiles were analyzed using flow cytometry. Comparing with untreated control (15% of cells in G2/M phase), treating cells with either 3.5 μM PENAO or 20 mM DCA resulted in a slight but significant (p < 0.05) increase in the proportion of cells (23% and 22% respectively) in the G2/M phase of the cell cycle (Figure 1E). A combination of both 3.5 μM PENAO and 20 mM DCA significantly affected (p < 0.01) the cell cycle dynamics with 37% cells found in G2/M phase (Figure 1E). Coinciding with the observed increase in the number of cells in G2/M arrest, the percentage population of cells in the S phase significantly decreased (p < 0.05) from 30% in the untreated to 15% in the combination treated group (Figure 1E). Similar trend of cell cycle distribution change in BAH1 cells also occurred after treated with PENAO, DCA and their combination (Figure 1E).

### DCA enhances the cytotoxicity of PENAO in GBM cells

**Figure 2:**
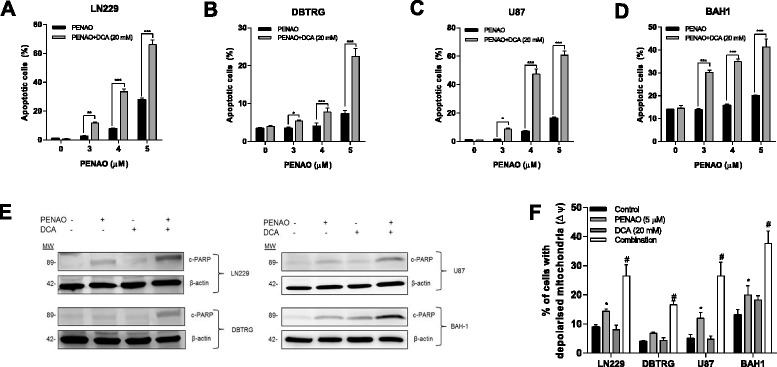
**DCA enhanced the cytotoxicity of PENAO in GBM cells. (A, B, C and D)** Percentage of apoptotic cells (Annexin V positive) after 24 hr treatment with PENAO (3, 4 and 5 μM), 20 mM DCA, and their combination. **(E)** The expression of cleaved PARP (c-PARP) in GBM cells (LN229, DBTRG, U87 and BAH1) after treatments with 5 μM PENAO, 20 mM DCA and their combination. Western blots presented are representative of 3 independent experiments. **(F)** The percentage of GBM cells (LN229, DBTRG, U87 and BAH1) with depolarized mitochondria after 16 hr treatment with PENAO, DCA and the combination. Results are presented as means ± s.d. of three experiments. *p < 0.05, **p < 0.01, ***p < 0.001, #p < 0.01 vs PENAO.

Further experiments were performed on GBM cells (U87, DBTRG, LN229 and BAH1) to determine whether the increased effect of PENAO by DCA was due to increased induction of apoptosis. Apoptosis was measured by the Annexin V staining. As a single drug, 20 mM DCA alone did not induce apoptosis in all four cell lines; however PENAO was able to induce apoptosis dose-dependently (Figure 2A,B,C and D). Furthermore, treating cells with the combination led to significant increases in the proportion of apoptotic cells compared to PENAO alone (Figure 2A,B,C, and D). Apoptotic cell death was further confirmed by the cleavage of PARP (c-PARP) (Figure 2E) and the depolarization of mitochondrial membrane potential (Figure 2F) in the 4 GBM cell lines employed, indicating that the cytotoxicity of PENAO was enhanced by DCA via the mitochondria-mediated apoptotic pathway.

### The oxidative stress plays a prominent role in the cytotoxicity of PENAO-DCA combination

**Figure 3:**
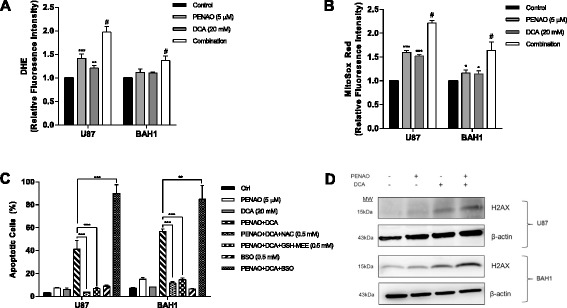
**The oxidative stress plays a prominent role in the cytotoxicity of PENAO-DCA combination. (A)** Cellular ROS levels in U87 and BAH1 cells after 6 hr treatment with 5 μM PENAO, 20 mM DCA and their combination. **(B)** Mitochondrial ROS levels in U87 and BAH1 cells after 6 hr treatment with PENAO, DCA and combination. **(C)** Percentage of apoptotic cells (Annexin V positive) after 24 hr PENAO-DCA treatment +/− 0.5 mM NAC or +/−0.5 mM GSH-MEE or +/− 0.5 mM BSO. **(D)** The expression of phosphorylated histone (γ-H2AX) after 24 hr treatment with PENAO, DCA and PENAO-DCA combination (PENAO and DCA concentrations as in A). Western blots presented are representative of 3 independent experiments. Results are presented as means ± s.d. of three experiments. *p < 0.05, **p < 0.01, ***p < 0.001, #p < 0.01 vs PENAO.

The mechanisms leading to enhanced apoptosis were further examined in U87 and BAH1 cells. Increased oxidative stress contributes to apoptotic cell death. DCA increases oxidative stress by directing pyruvate into mitochondria, increasing electron transport chain activity and thus generating more reactive oxygen species (ROS) [15,17,21]. This increased oxidative stress could potentially sensitize cancer cells towards apoptosis [29]. PENAO has been shown to induces apoptosis in several cancer cell lines in culture via the induction of oxidative stress [5,25,26]. We therefore investigated whether the cytotoxicity of PENAO-DCA combination was regulated by the change of redox system. GBM cells (U87 and BAH1) were treated with 5 μM PENAO alone, 20 mM DCA alone and a combination of PENAO and DCA for 6 hr. Total cellular ROS levels and mitochondrial superoxide were evaluated by using DHE and MitoSOX Red, respectively. As single agents, both PENAO and DCA slightly increased mitochondrial and total cellular ROS levels in both U87 and BAH1 cells. However, the total cellular and mitochondrial ROS induced by the combination are significantly higher than those induced by each single agent (p < 0.01) (Figure 3A,B). Specifically, the combination of PENAO and DCA resulted in 1.5 ~ 2-fold increases in the fluorescence of DHE and 1.7 ~ 2.2-fold increases in MitoSOX Red fluorescence when compared to untreated control (Figure 3A,B).

To further investigate the regulation of PENAO-DCA cytotoxicity by the redox system, the antioxidant, NAC, and an inhibitor of glutathione (GSH) synthesis, BSO, were employed. Treatment of GBM cells with 0.5 mM NAC significantly inhibited cell death induced by PENAO-DCA treatment (p < 0.001) (Figure 3C). To directly confirm whether reduced GSH played a role in the inhibition of PENAO-DCA induced cytotoxicity, cell-permeable form of GSH (GSH-MEE) was employed. Addition of GSH-MEE also significantly revoked cell death in both U87 and BAH1 cells, compared to those treated with PENAO-DCA combination (p < 0.001) (Figure 3C). In contrast, depletion of glutathione pool by BSO aggravated the cytotoxicity induced by PENAO-DCA combination significantly (Figure 3C). BSO alone (0.5 mM) did not show any effect on GBM cells. However, combined treatment of PENAO-DCA and BSO significantly increased the number of apoptotic cells by 1.3 ~ 2-fold compared to the combination of PENAO and DCA (Figure 3C). Taken together, these findings indicate GSH-regulated redox change plays a pivotal role in the cytotoxic effect of PENAO-DCA treatment.

Increased level of superoxide and other ROS can cause severe damage to cellular macromolecules, especially DNA. Oxidative damage to DNA can lead to double strand breaks and phosphorylation of histone (γ-H2AX), a hallmark of DNA damage [30]. Because the PENAO-DCA combination induced higher cellular and mitochondrial levels of ROS than each drug alone, a further experiment was conducted to investigate the change of γ-H2AX level after treatment of PENAO and DCA. Consistently, the co-treatment of PENAO and DCA induced higher levels of γ-H2AX compared to single treatments (Figure 3D). These data strongly support that the effect of PENAO-DCA combination was exerted through the redox stress.

### DCA treatment inhibits MRP1 expression in GBM cells

**Figure 4:**
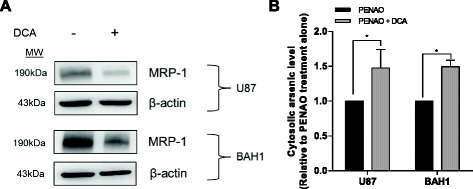
**DCA treatment inhibits MRP1 expression, thereby increasing the cytosolic accumulation of PENAO in GBM cells. (A)** The expression of MRP1 in GBM cells (U87 and BAH1 cells) after 24 hr treatment with 20 mM DCA. Western blots presented are representative of 3 independent experiments. **(B)** Cellular levels of PENAO in U87 and BAH1 cells with/without 24 hr pre-treatment with 20 mM DCA. Results are presented as means ± s.d. of triplicate measurements. *p < 0.05.

A recent study reported DCA treatment led to an alteration in the multidrug resistance protein (MDR) phenotype of tumor cells (inhibition of multidrug resistant protein 1, MRP1), thereby enhancing the effectiveness of cisplatin in a Dalton's lymphoma mouse model [31]. MRP1 is known to blunt the effect of PENAO by exporting it from cytosol [25]. Thus we questioned whether the enhanced cytotoxicity of PENAO by DCA treatment was due to the inhibition of MRP1 expression in GBM cells, which in turn increases the accumulation of PENAO in the cytosol. As expected, western blot successfully demonstrated that the expression of MRP1 in both U87 and BAH1 cells was down-regulated after 24 hr DCA treatment (Figure 4A). Next, the cytosolic level of PENAO was determined by mass spectrometry showing the level of arsenical atom, a surrogate measure for PENAO levels, accumulated in cytosol. After 24 hr pre-treatment with 20 mM DCA, the level of PENAO accumulation was ~1.5-fold higher than that without DCA pre-treatment (Figure 4B).

### DCA promotes oxidative phosphorylation through inhibiting glycolysis and attenuates PENAO-induced acid production

**Figure 5:**
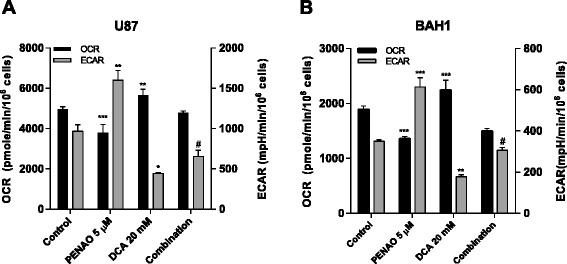
**DCA promotes oxidative phosphorylation through inhibiting glycolysis and attenuates PENAO-induced acid production.** Two metabolic parameters, OCR and ECAR, were measured in **(A)** U87 cells and **(B)** BAH1 cells after 24 hr treatment with 5 μM PENAO, 20 mM DCA and the combination. Results are representative of three independent experiments and presented as means ± s.d. of 3–5 measurements. *p < 0.05, **p < 0.01, ***p < 0.001, #p < 0.001 vs PENAO.

To confirm the anti-metabolic effect of PENAO-DCA combination, two metabolic parameters, oxygen consumption rate (OCR) and extracellular acidification rate (ECAR), were measured using a Seahorse XF24 analyzer. PENAO, as a mitochondrial inhibitor, is expected to inhibit oxygen consumption whilst stimulate acid production. In contrast, DCA is expected to redirect pyruvate to mitochondrial metabolism at the expense of acidification. To examine this, the bioenergetic profiles were obtained under the same experimental conditions following 24 hr PENAO and/or DCA treatments. As shown in Figure 5A, treatment of U87 cells with 5 μM PENAO resulted in a significant decrease in OCR (p < 0.001) as well as a substantial increase in ECAR level (p < 0.01) compared to untreated control. In contrast, DCA treatment led to a slight but significant increase in OCR (p < 0.01) and a sharp decrease (p < 0.05) in ECAR levels compared to control cells (Figure 5A). When treated with the combination, the PENAO-induced acid production was largely eliminated by the addition of DCA (Figure 5A). Similar metabolic profile of BAH1 cells treated with the PENAO, DCA and the combination therapy further confirmed results we observed in U87 cells (Figure 5B).

## Discussion

A classic bioenergetic adaptation of cancer cells is the metabolic shift from OXPHOS to glycolysis regardless of oxygen availability, a phenomenon termed the Warburg Effect [2]. The high glycolytic metabolism of GBM makes it an attractive target of anti-glycolytic therapy. Although several agents that specifically target glycolysis have been used as effective anticancer agents *in vitro* and *in vivo* [3-5], this approach has yielded very few positive results in clinical trials. In particular, recent studies of human orthotopic gliomas in mice and *in situ* demonstrated the Warburg Effect may not necessarily be the case in brain tumors, where mitochondrial OXPHOS appears to be more important for the glucose metabolism and chemoresistance [32,33]. Although it is still unsettled as to whether cancer cells in the brain preferentially catabolize glucose via aerobic glycolysis or OXPHOS, it has been proposed that tumor cells can utilize either glycolysis or OXPHOS due to their plasticity of metabolic circuit, particularly when one or the other pathway is blocked therapeutically [34]. Therefore, a better therapeutic strategy may arise if dual-blockade of bioenergetic metabolism is applied.

Previous studies have demonstrated that selective targeting of mitochondrial metabolism of tumor cells synergistically aggravated cytotoxicity in tumor cells treated with glycolytic inhibitors [35,36]. Both PENAO and DCA are currently being evaluated in clinical trials in the treatment of cancer that target different aspects of the cancer metabolic phenotype [26,37]. In the current study, we demonstrated that PENAO, a novel mitochondria-targeted agent, is effective at low micromolar concentrations against a range of GBM cell lines, consisting of immortalized and patient-derived GBM cell lines. The anti-tumor activity of DCA has been shown *in vitro*, *in vivo*, and in a small cohort of GBM patients [13,15,17,38-40]. However, it has been noticed that there is a discordance in DCA efficacy when it was tested *in vitro* and *in vivo* [16]. In the present study, the IC_50_ values of DCA are relatively high (≥20 mM, 72 hr) to halt the proliferation of GBM cells in culture, which is consistent with most of the studies *in vitro* [13,23,24,39-43]. On the other hand, *in vivo* studies testing DCA on pre-clinical rodent models with different types of malignancy demonstrated encouraging results with effective doses of 50–200 mg/kg/day [39,40,44], which translates into approximately 13 kg/kg/day in human resulting in the serum level of 0.5-1 mM [15,40]. Therefore, the *in vivo* achievable concentration (0.5-1 mM) may not necessarily be correlated with the supraphamacological level (5–50 mM) tested *in vitro* for this anti-metabolic compound.

When these cells were treated with the combination of PENAO and DCA, cell proliferation was further inhibited as opposed to each treatment alone. This finding was also supported by the analysis of cell cycle distribution showing the significant increase of cell proportions in the G2/M phase as well as the obvious decreases in the S phase. As cancer cells are most sensitive to radiation therapy in the G2/M phase of the cell cycle whilst most resistance in the S phase [45-47], these findings suggest that PENAO-DCA treatment may have the potential in sensitizing GBM cells to ionizing irradiation. In addition, we also observed that 20 mM DCA alone failed to induce effective apoptosis, but it enhanced PENAO-induced apoptosis when co-treatment was administered. PENAO-DCA combination caused a synergistic cytotoxicity selectively in GBM cells whilst sparing toxicity on non-cancerous cells, indicating a therapeutic window between normal and cancer cells exists. The mode of cytotoxic action of PENAO is correlated with enhanced mitochondrial production of ROS, depolarization of mitochondrial membrane potential and inhibition of OXPHOS all leading to mitochondria-mediated apoptotic cell death [48]. DCA was combined with PENAO, as we proposed that by reversing the glycolytic phenotype with DCA and directing more pyruvate into mitochondrial OXPHOS, simultaneous targeting of the mitochondria with PENAO would be more effective than PENAO alone. Our findings confirmed this hypothesis by showing that DCA worked synergistically with PENAO as evidenced from the significant increases in proliferation and G2/M cell cycle arrest, impaired clonogenicity, ROS production, DNA damage, depolarization of mitochondrial membrane potential, and apoptosis compared to each treatment alone. The general principles of this dual-targeting strategy are supported by previous literature which has demonstrated that cells with mitochondrial defects displayed higher sensitivity to the cytostatic effects of DCA [10,21,49]. In addition, DCA has been demonstrated to potentiate the anticancer effect of several drugs, such as sulindac, 5-FU and omeprazole, through ROS production, loss of mitochondrial membrane potential and apoptotic cell death [43,50,51]. The involvement of ROS in apoptosis mediated by DCA was demonstrated both by reducing apoptotic cells with anti-oxidant such as NAC, GSH-MEE and by augmenting cell death with glutathione depletion [41,42,50]. These findings were also corroborated in this study by the fact that the cytotoxicity of PENAO-DCA combination was clearly regulated by GSH-mediated redox change when NAC, GSH-MEE and BSO were added. The mode of action of this combination further suggests it may serve as a potent chemotherapeutic sensitizer to improve the efficacy of chemotherapeutic reagents used in the current standard of care, e.g. temozolomide, by boosting the generation of ROS in GBM cells.

Interestingly, a recent study reported DCA treatment led to an inhibition of MRP1 expression, thereby enhancing the effectiveness of cisplatin in a Dalton's lymphoma mouse model [31]. MRP1 is known to be well expressed in GBM cells [52] and to blunt the effect of PENAO by exporting it out of the cytosol [25]. Therefore, we examined the expression of MRP1 in GBM cells after DCA treatment. Consistently, we found pre-treatment with DCA reduced MRP1 expression in GBM cells, thereby increasing/retaining the cytosolic level of PENAO compared to that treated with PENAO alone (without DCA pre-treatment). This finding revealed another underlying mechanism of enhanced cytotoxicity achieved by the combination of PENAO and DCA. Metabolically, the addition of DCA largely abrogated PENAO-induced glycolytic rate that would provide cancer cells with numerous advantages via the Warburg effect [53]. In this respect, PENAO-DCA combination provides a dual therapeutic advantage as DCA enhances the cytotoxicity of PENAO to GBM cells through a mechanism involving oxidative stress while simultaneously lowering acid production induced by PENAO. Notably, recent findings have indicated that a metabolic shift to glycolysis occurred in GBM cells during anti-angiogenic therapy with bevacizumab, which was thought to be associated with resistance to anti-angiogenic therapy and enhanced tumor cell invasion [54]. Most importantly, thus far there is no effective treatment for recurrent GBM patients who progress following bevacizumab treatment. Reversal of the bevacizumab-induced shift in glucose metabolism using DCA has been shown to effectively inhibit the neoplastic growth of GBM *in vivo* [40]. Therefore, adjuvant therapy with drugs targeting both glycolytic and mitochondrial glucose metabolism could be more beneficial in the anti-angiogenic therapy for GBM.

## Conclusions

Our findings provide the first *in vitro* proof of concept that dual-targeting of glucose metabolism is an effective therapeutic approach to eradicate GBM cells. This combinatorial strategy halts proliferation, elevates ROS production, depolarizes mitochondrial membrane potential, and induces apoptosis in GBM cells. In addition, DCA inhibits the expression of MRP1, which in turn increases the cytosolic accumulation of PENAO. Moreover, DCA attenuates the acid production induced by PENAO treatment metabolically. Taken together, the findings of this study warrant further evaluation of this combination *in vivo*. As PENAO-DCA combination has the potential to be beneficial in the context of radiotherapy (G2/M arrest) and temozolomide (ROS production), this dual-targeting therapy might serve as a novel therapy for GBM.

## Abbreviations

GBM: Glioblastoma
PENAO: 4-(N-(S-penicillaminylacetyl)amino) phenylarsonous acid
DCA: Dichloroacetate
GSH: Glutathione
MDR: Multidrug resistance
OXPHOS: Oxidative phosphorylation
PDK: Pyruvate dehydrogenase kinase
ETC: Electron transport chain
ANT: Adenine nucleotide translocase
MPTP: Mitochondrial permeability transition pore
GSAO: 4-(N-(S-glutathionylacetyl)amino) phenylarsonous acid
MRP: Multidrug resistant protein
NAC: N-acetyl-L-cysteine
GSH-MEE: Glutathione reduced ethyl ester
BSO: Buthionine sulphoximine
PBS: Phosphate-buffered saline
HBSS: Hank’s balanced salt solution
PI: Propidium iodide
DHE: Dihydroethdium
c-PARP: Cleaved Poly (ADP-ribose) polymerase
ROS: Reactive oxygen species
OCR: Oxygen consumption rate
ECAR: Extracellular acidification rate
IC_50_: The half-maximal inhibitory concentration
